# *catena*-Poly[[diaqua­zinc(II)]-μ-4,4′-(methyl­enedioxy)­dibenzoato]

**DOI:** 10.1107/S1600536809006965

**Published:** 2009-03-06

**Authors:** Lei Xu, Yingna Guo, Xing Yuan

**Affiliations:** a College of Urban and Environmental Sciences, Northeast Normal University, Changchun 130024, People’s Republic of China; b School of Chemistry and Environmental Engineering, Changchun University of Science and Technology, Changchun 130022, People’s Republic of China

## Abstract

In the title complex, [Zn(C_15_H_10_O_6_)(H_2_O)_2_]_*n*_, the Zn^II^ atom is located on a twofold rotation axis and exhibits a distorted tetrahedral coordination environment defined by two O atoms from two 4,4′-(methyl­enedioxy)­dibenzoate ligands and two O atoms from two coordinated water mol­ecules. In the crystal structure, mol­ecules are linked into a three-dimensional framework by O—H⋯O hydrogen bonds and C—H⋯π inter­actions.

## Related literature

For the potential properties and structural topologies of metal-organic complexes involving polycarboxyl­ate ligands, see: Chen & Liu (2002 ▶); Han *et al.* (2009 ▶); Li *et al.* (2007 ▶).
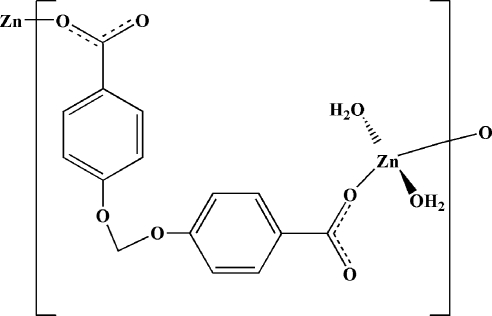


## Experimental

### 

#### Crystal data

[Zn(C_15_H_10_O_6_)(H_2_O)_2_]
*M* *_r_* = 387.63
Monoclinic, 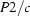

*a* = 13.496 (1) Å
*b* = 4.931 (1) Å
*c* = 12.357 (1) Å
β = 113.352 (1)°
*V* = 755.0 (2) Å^3^
*Z* = 2
Mo *K*α radiation
μ = 1.67 mm^−1^
*T* = 293 K
0.21 × 0.19 × 0.15 mm

#### Data collection

Rigaku R-AXIS RAPID diffractometer
Absorption correction: multi-scan (*ABSCOR*; Higashi, 1995 ▶) *T*_min_ = 0.707, *T*_max_ = 0.780
4318 measured reflections
1696 independent reflections
1461 reflections with *I* > 2σ(*I*)
*R*_int_ = 0.027

#### Refinement

*R*[*F* ^2^ > 2σ(*F* ^2^)] = 0.032
*wR*(*F* ^2^) = 0.075
*S* = 1.06
1696 reflections
118 parameters
H atoms treated by a mixture of independent and constrained refinement
Δρ_max_ = 0.25 e Å^−3^
Δρ_min_ = −0.30 e Å^−3^

### 

Data collection: *PROCESS-AUTO* (Rigaku, 1998 ▶); cell refinement: *PROCESS-AUTO*; data reduction: *PROCESS-AUTO*; program(s) used to solve structure: *SHELXS97* (Sheldrick, 2008 ▶); program(s) used to refine structure: *SHELXL97* (Sheldrick, 2008 ▶); molecular graphics: *SHELXTL-Plus* (Sheldrick, 2008 ▶); software used to prepare material for publication: *SHELXL97*.

## Supplementary Material

Crystal structure: contains datablocks I, global. DOI: 10.1107/S1600536809006965/at2728sup1.cif

Structure factors: contains datablocks I. DOI: 10.1107/S1600536809006965/at2728Isup2.hkl

Additional supplementary materials:  crystallographic information; 3D view; checkCIF report

## Figures and Tables

**Table:** 

Zn1—O1	1.9582 (18)
Zn1—O4	1.975 (2)

**Table:** 

O1—Zn1—O1^i^	102.26 (11)
O1—Zn1—O4	99.87 (9)
O1^i^—Zn1—O4	132.40 (8)
O4—Zn1—O4^i^	95.27 (13)

Symmetry code: (i) 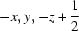.

**Table 2:** Hydrogen-bond geometry (Å, °)

*D*—H⋯*A*	*D*—H	H⋯*A*	*D*⋯*A*	*D*—H⋯*A*
O4—H1⋯O1^ii^	0.74 (3)	2.13 (3)	2.851 (3)	167 (3)
O4—H2⋯O2^iii^	0.88 (4)	1.78 (4)	2.657 (3)	177 (4)
C8—H8*A*⋯*Cg*3^iv^	0.97	2.97	3.741 (3)	137
C8—H8*B*⋯*Cg*3^v^	0.97	2.97	3.741 (3)	137

Symmetry codes: (ii) 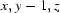; (iii) 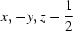; (iv) 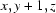; (v) 
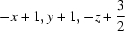
. *Cg*3 is the centroid of the C2–C7 benzene ring.

**Fig. 1.**
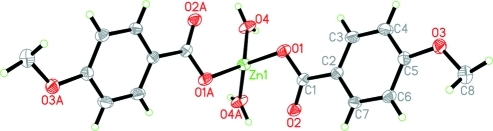
View of the local coordination of Zn(II) with the atomic numbering scheme. Displacement ellipsoids are drawn at the 30% probability level.

**Fig. 2.**
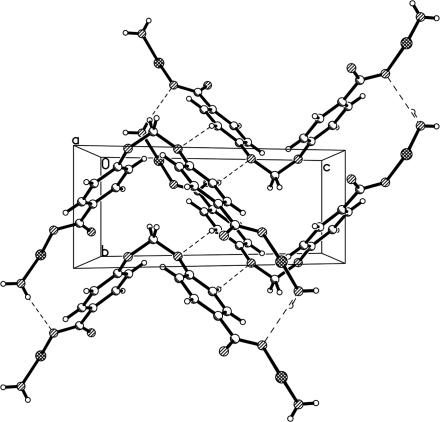
A packing diagram for the three-dimensional supramolecular framework via O—H···O interactions. The view direction is parallel to the a axis. Hydrogen bonds are indicated by dashed lines.

## supplementary crystallographic information

### Comment

Recently, the area of metal-organic framework materials has become one of the intense research activity for their fascinating structural diversities and potential applications in catalysis, nonlinear optics and molecular sensing. As an important family of multidentate O-donor ligands, organic aromatic polycarboxylate ligands have been extensively employed in the preparation of metal-organic complexes because of their potential properties and intriguing structural topologies (Han *et al.*, 2009; Li *et al.*, 2007; Chen *et al.*, 2002). Herein, we report the structure of the title complex with bis(4-benzoateoxyl)methane and zinc, [Zn(C_15_H_10_O_6_)(H_2_O)_2_] (I).

Single-crystal X-ray diffraction analyses revealed Zn(II) is tetra-coordinated and exhibits tetrahedral coordination environment supplied by two bis(4-benzoateoxyl)methane O atoms and two water molecules (Fig. 1). The Zn—O bond lengthes are in the normal range (Table 1). The bis(4-benzoateoxyl)methane ligand adopts bidentate coordinated modes and bond with two zinc ions to form a chain. Adjacent chains are linked by O—H···O hydrogen bonds and C—H···π interactions into a three-dimensional supramolecular network structure (Fig. 2, Table 2).

### Experimental

Zinc(II) acetate dihydrate (0.066 g, 0.3 mol), bis(4-benzoateoxyl)methane (0.058 g, 0.2 mmol), sodium hydroxide (0.016 g, 0.4 mmol) and water (14 ml) were placed in a 23 ml Teflon-lined autoclave, and the autoclave was heated at 423 K for 3 d. After cooling slowly to room temperature at a rate of 10 K h^-1^, colourless crystals of (I) were obtained.

### Refinement

C-bound H atoms were treated as riding, with C—H = 0.93 and 0.97Å and *U*_iso_(H) = 1.2 times *U*_eq_(C). O-bound H atoms were located in a difference Fourier map and refined as riding in their as-found relative positions; *U*_iso_(H) = 1.5*U*_eq_(O).

### Crystal data

**Table:**

[Zn(C_15_H_10_O_6_)(H_2_O)_2_]	*F*(000) = 396
*M**_r_* = 387.63	*D*_x_ = 1.705 Mg m^−^^3^
Monoclinic, *P*2/*c*	Mo *K*α radiation, λ = 0.71069 Å
Hall symbol: -P 2yc	Cell parameters from 3185 reflections
*a* = 13.496 (1) Å	θ = 2.1–27.4°
*b* = 4.931 (1) Å	µ = 1.67 mm^−^^1^
*c* = 12.357 (1) Å	*T* = 293 K
β = 113.352 (1)°	Block, colourless
*V* = 755.0 (2) Å^3^	0.21 × 0.19 × 0.15 mm
*Z* = 2	

### Data collection

**Table:**

Rigaku R-AXIS RAPID diffractometer	1696 independent reflections
Radiation source: fine-focus sealed tube	1461 reflections with *I* > 2σ(*I*)
graphite	*R*_int_ = 0.027
Detector resolution: 10 pixels mm^-1^	θ_max_ = 27.5°, θ_min_ = 3.3°
ω scans	*h* = −10→17
Absorption correction: multi-scan (*ABSCOR*; Higashi, 1995)	*k* = −6→5
*T*_min_ = 0.707, *T*_max_ = 0.780	*l* = −15→15
4318 measured reflections	

### Refinement

**Table:**

Refinement on *F*^2^	Primary atom site location: structure-invariant direct methods
Least-squares matrix: full	Secondary atom site location: difference Fourier map
*R*[*F*^2^ > 2σ(*F*^2^)] = 0.032	Hydrogen site location: inferred from neighbouring sites
*wR*(*F*^2^) = 0.075	H atoms treated by a mixture of independent and constrained refinement
*S* = 1.06	*w* = 1/[σ^2^(*F*_o_^2^) + (0.0304*P*)^2^ + 0.2993*P*] where *P* = (*F*_o_^2^ + 2*F*_c_^2^)/3
1696 reflections	(Δ/σ)_max_ < 0.001
118 parameters	Δρ_max_ = 0.25 e Å^−^^3^
0 restraints	Δρ_min_ = −0.30 e Å^−^^3^

### Special details

**Table:**

Geometry. All e.s.d.'s (except the e.s.d. in the dihedral angle between two l.s. planes) are estimated using the full covariance matrix. The cell e.s.d.'s are taken into account individually in the estimation of e.s.d.'s in distances, angles and torsion angles; correlations between e.s.d.'s in cell parameters are only used when they are defined by crystal symmetry. An approximate (isotropic) treatment of cell e.s.d.'s is used for estimating e.s.d.'s involving l.s. planes.
Refinement. Refinement of *F*^2^ against ALL reflections. The weighted *R*-factor *wR* and goodness of fit *S* are based on *F*^2^, conventional *R*-factors *R* are based on *F*, with *F* set to zero for negative *F*^2^. The threshold expression of *F*^2^ > σ(*F*^2^) is used only for calculating *R*-factors(gt) *etc*. and is not relevant to the choice of reflections for refinement. *R*-factors based on *F*^2^ are statistically about twice as large as those based on *F*, and *R*- factors based on ALL data will be even larger.

### Fractional atomic coordinates and isotropic or equivalent isotropic displacement parameters (Å^2^)

**Table:**

	*x*	*y*	*z*	*U*_iso_*/*U*_eq_	Occ. (<1)
Zn1	0.0000	0.02532 (7)	0.2500	0.03611 (14)	
O3	0.48509 (13)	1.0388 (3)	0.65056 (14)	0.0430 (4)	
O4	0.06484 (17)	−0.2446 (4)	0.17945 (17)	0.0484 (5)	
O2	0.07014 (13)	0.2515 (3)	0.46679 (14)	0.0420 (4)	
O1	0.12200 (13)	0.2746 (3)	0.31887 (13)	0.0406 (4)	
C5	0.39531 (18)	0.8759 (5)	0.60267 (19)	0.0351 (5)	
C7	0.22564 (18)	0.6856 (4)	0.58165 (19)	0.0356 (5)	
H7	0.1685	0.6754	0.6055	0.043*	
C1	0.13275 (18)	0.3401 (4)	0.42419 (18)	0.0331 (5)	
C8	0.5000	1.1980 (7)	0.7500	0.0451 (8)	
H8A	0.4375	1.3138	0.7330	0.054*	0.50
H8B	0.5625	1.3138	0.7670	0.054*	0.50
C2	0.22362 (18)	0.5269 (4)	0.48823 (19)	0.0328 (5)	
C6	0.31099 (19)	0.8591 (5)	0.6403 (2)	0.0379 (5)	
H6	0.3119	0.9624	0.7037	0.046*	
C3	0.3096 (2)	0.5481 (5)	0.4528 (2)	0.0434 (6)	
H3	0.3092	0.4453	0.3896	0.052*	
C4	0.3942 (2)	0.7180 (5)	0.5100 (2)	0.0447 (6)	
H4	0.4515	0.7273	0.4863	0.054*	
H2	0.069 (3)	−0.244 (7)	0.110 (3)	0.100 (13)*	
H1	0.085 (2)	−0.374 (6)	0.210 (2)	0.046 (8)*	

### Atomic displacement parameters (Å^2^)

**Table:**

	*U*^11^	*U*^22^	*U*^33^	*U*^12^	*U*^13^	*U*^23^
Zn1	0.0455 (2)	0.0276 (2)	0.0380 (2)	0.000	0.01943 (18)	0.000
O3	0.0416 (9)	0.0491 (10)	0.0364 (9)	−0.0115 (8)	0.0132 (7)	0.0005 (7)
O4	0.0832 (14)	0.0311 (10)	0.0397 (10)	0.0128 (9)	0.0338 (10)	0.0040 (8)
O2	0.0471 (9)	0.0448 (9)	0.0359 (9)	−0.0096 (8)	0.0183 (7)	0.0003 (7)
O1	0.0538 (10)	0.0371 (9)	0.0338 (9)	−0.0057 (8)	0.0205 (8)	−0.0074 (7)
C5	0.0361 (12)	0.0332 (11)	0.0337 (12)	−0.0026 (10)	0.0114 (10)	0.0052 (10)
C7	0.0367 (12)	0.0370 (12)	0.0370 (12)	−0.0021 (10)	0.0189 (10)	−0.0009 (10)
C1	0.0404 (12)	0.0269 (10)	0.0293 (11)	0.0039 (9)	0.0110 (10)	0.0010 (9)
C8	0.045 (2)	0.0317 (17)	0.051 (2)	0.000	0.0109 (16)	0.000
C2	0.0379 (12)	0.0304 (11)	0.0301 (11)	−0.0009 (9)	0.0135 (9)	0.0015 (9)
C6	0.0445 (13)	0.0378 (12)	0.0338 (12)	−0.0033 (10)	0.0179 (11)	−0.0055 (10)
C3	0.0488 (14)	0.0483 (14)	0.0398 (13)	−0.0055 (12)	0.0246 (12)	−0.0078 (11)
C4	0.0447 (14)	0.0506 (14)	0.0483 (14)	−0.0037 (12)	0.0285 (12)	−0.0033 (12)

### Geometric parameters (Å, °)

**Table:**

Zn1—O1	1.9582 (18)	C7—C2	1.386 (3)
Zn1—O1^i^	1.9582 (18)	C7—C6	1.387 (3)
Zn1—O4	1.975 (2)	C7—H7	0.9300
Zn1—O4^i^	1.975 (2)	C1—C2	1.487 (3)
O3—C5	1.377 (3)	C8—O3^ii^	1.404 (2)
O3—C8	1.404 (2)	C8—H8A	0.9700
O4—H2	0.88 (4)	C8—H8B	0.9700
O4—H1	0.74 (3)	C2—C3	1.396 (3)
O2—C1	1.239 (3)	C6—H6	0.9300
O1—C1	1.292 (3)	C3—C4	1.366 (3)
C5—C4	1.380 (3)	C3—H3	0.9300
C5—C6	1.392 (3)	C4—H4	0.9300
			
O1—Zn1—O1^i^	102.26 (11)	O1—C1—C2	115.52 (19)
O1—Zn1—O4	99.87 (9)	O3—C8—O3^ii^	112.0 (3)
O1^i^—Zn1—O4	132.40 (8)	O3—C8—H8A	109.2
O1—Zn1—O4^i^	132.40 (8)	O3^ii^—C8—H8A	109.2
O1^i^—Zn1—O4^i^	99.87 (9)	O3—C8—H8B	109.2
O4—Zn1—O4^i^	95.27 (13)	O3^ii^—C8—H8B	109.2
C5—O3—C8	119.93 (16)	H8A—C8—H8B	107.9
Zn1—O4—H2	130 (2)	C7—C2—C3	118.3 (2)
Zn1—O4—H1	119 (2)	C7—C2—C1	122.2 (2)
H2—O4—H1	111 (3)	C3—C2—C1	119.5 (2)
C1—O1—Zn1	109.52 (14)	C7—C6—C5	118.8 (2)
O3—C5—C4	113.8 (2)	C7—C6—H6	120.6
O3—C5—C6	126.0 (2)	C5—C6—H6	120.6
C4—C5—C6	120.2 (2)	C4—C3—C2	120.8 (2)
C2—C7—C6	121.5 (2)	C4—C3—H3	119.6
C2—C7—H7	119.3	C2—C3—H3	119.6
C6—C7—H7	119.3	C3—C4—C5	120.4 (2)
O2—C1—O1	121.1 (2)	C3—C4—H4	119.8
O2—C1—C2	123.4 (2)	C5—C4—H4	119.8
			
O1^i^—Zn1—O1—C1	86.84 (14)	O1—C1—C2—C7	158.0 (2)
O4—Zn1—O1—C1	−135.58 (15)	O2—C1—C2—C3	159.3 (2)
O4^i^—Zn1—O1—C1	−29.01 (18)	O1—C1—C2—C3	−21.1 (3)
C8—O3—C5—C4	−176.2 (2)	C2—C7—C6—C5	1.0 (3)
C8—O3—C5—C6	3.1 (3)	O3—C5—C6—C7	179.5 (2)
Zn1—O1—C1—O2	−1.0 (3)	C4—C5—C6—C7	−1.2 (3)
Zn1—O1—C1—C2	179.28 (14)	C7—C2—C3—C4	0.9 (4)
C5—O3—C8—O3^ii^	64.94 (16)	C1—C2—C3—C4	−180.0 (2)
C6—C7—C2—C3	−0.9 (3)	C2—C3—C4—C5	−1.2 (4)
C6—C7—C2—C1	−179.9 (2)	O3—C5—C4—C3	−179.3 (2)
O2—C1—C2—C7	−21.7 (3)	C6—C5—C4—C3	1.3 (4)

Symmetry codes: (i) −*x*, *y*, −*z*+1/2; (ii) −*x*+1, *y*, −*z*+3/2.

### Hydrogen-bond geometry (Å, °)

**Table:**

*D*—H···*A*	*D*—H	H···*A*	*D*···*A*	*D*—H···*A*
O4—H1···O1^iii^	0.74 (3)	2.13 (3)	2.851 (3)	167 (3)
O4—H2···O2^iv^	0.88 (4)	1.78 (4)	2.657 (3)	177 (4)
C8—H8A···Cg3^v^	0.97	2.97	3.741 (3)	137
C8—H8B···Cg3^vi^	0.97	2.97	3.741 (3)	137

Symmetry codes: (iii) *x*, *y*−1, *z*; (iv) *x*, −*y*, *z*−1/2; (v) *x*, *y*+1, *z*; (vi) −*x*+1, *y*+1, −*z*+3/2.
